# An enquiry into my use of supervised clinical assessments in the supervision of junior trainees

**DOI:** 10.1192/bjo.2021.374

**Published:** 2021-06-18

**Authors:** Yuan Choo

**Affiliations:** Dorset Healthcare University NHS Foundation Trust

## Abstract

**Aims:**

As a particular example of action research, to enquire into my use of Assessments of Clinical Expertise in my supervision of junior trainees, with the intention of further developing my own practice as an educator.

**Background:**

Work-Place Based Assessments (WPBAs) play an established role currently in the assessment of trainee doctors(tenCate, 2017). In psychiatry, supervised clinical assessments(ACE/mini-ACE) assess a trainee's proficiency in various areas. As part of my PGCert in Medical Education, I was inspired to examine how I conduct and utilise this form of assessment, and indeed the underpinning values and beliefs, about learning, and developing professional wisdom.

**Method:**

This enquiry was situated within the interpretivist tradition. I interrogated my views about the epistemology of knowledge, and how they had changed from pre-university. I made clear my influences from Coles (Fish & Coles, 1998) on professional practice. I investigated my values in performing an assessment, comparing them to those of the wider community. I examined the literature on the validity of this as a tool. I then performed an assessment of a junior, with a consultant observing, before interviewing them separately.

**Result:**

There has been a paradigm shift in how I view assessments, from pre-university in Singapore, to medical training in the UK. The history of WPBAs and the values espoused is intriguing. Consultants and experts may view assessments differently from trainees, but a core value of developing professional judgement is common.

In my interview with the consultant, there were themes around having a clear focus for an assessment, and provision of feedback; the rating scales and how they used them to stimulate feedback; and our shared values in performing an assessment. With the junior, the themes were around the delivery of feedback (including non-verbal), an appreciation of my encouraging self-reflection and understanding, and the observable values in my carrying out of the assessment, which could be compared to those of other assessors.

**Conclusion:**

WPBAs have their merits, and shortfalls. I am aware of my values and beliefs when utilising them, and have identified a plan to further develop my own practice. This case study is particular, but possibly not unique, in how WPBAs are used in medical education.

